# The use of Twitter by people with young-onset dementia: A qualitative analysis of narratives and identity formation in the age of social media

**DOI:** 10.1177/14713012211002410

**Published:** 2021-03-25

**Authors:** Catherine V Talbot, Siobhan T O’Dwyer, Linda Clare, Janet Heaton

**Affiliations:** Department of Psychology, 276175 Bournemouth University, UK; College of Medicine and Health, 171002 University of Exeter, UK; Division of Rural Health and Wellbeing, 7709 University of the Highlands and Islands, UK

**Keywords:** Alzheimer’s, communication, social media, tweet

## Abstract

A diagnosis of dementia in midlife can be challenging, causing losses or changes in a person’s identity. Narrative provides a means of reconstructing identity and can be communicated on social media. There has been initial evidence on the value of Twitter for people with dementia, but researchers have not yet directly engaged with users’ perspectives. We employed a narrative model of identity to examine why people with dementia use Twitter and what challenges they face. Interviews were conducted with 11 younger people with dementia and analysed thematically. Participants used Twitter to counter a loss of identity through community membership and by regaining a sense of purpose. They sought to redefine dementia identities by challenging stigma and campaigning for social change. The character limit of tweets facilitated narrative through which participants preserved their identities. These findings suggest that Twitter could be an important source of post-diagnostic support for people with young-onset dementia. However, there are some risks as Twitter was sometimes a hostile environment for individuals who did not present in a ‘typical’ manner, or faced technical difficulties because of their symptoms. In the future, platform developers could work with people with dementia to make Twitter more accessible for this group.

## Introduction

There are an estimated 42,000 people in the United Kingdom (UK) living with a dementia diagnosis made before the age of 65 years (i.e. young-onset dementia; Prince et al., 2014). Whilst a diagnosis of dementia can be difficult for anyone, those under the age of 65 years face distinct challenges. People with young-onset dementia tend to have more financial commitments and caregiving responsibilities than those diagnosed later in life (Greenwood & Smith, 2016; Johannessen & Möller, 2011), and a diagnosis can have a powerful impact on their sense of identity (Greenwood & Smith, 2016; Griffin et al., 2016; Harris & Keady, 2009; Spreadbury & Kipps, 2019). For example, people with young-onset dementia have reported changes and losses in their identities as workers, partners and parents, which they associated with their perceived intellect, self-esteem and self-efficacy (Griffin et al., 2016; Harris & Keady, 2009; Rabanal et al., 2018). As a result of these changes, people with young-onset dementia can withdraw from meaningful activities and experience a loss of purpose (Roach & Drummond, 2014; Johannessen & Möller, 2011).

The perceptions of others can also shape a person’s sense of identity. Dementia continues to be a stigmatised condition, whereby those with the diagnosis are frequently identified as ‘victims’ and ‘sufferers’ (Peel, 2014), and their symptoms are often portrayed negatively and unrealistically (Gerritsen et al., 2014). Swaffer (2015) argued that the negative language surrounding dementia influences not only societal responses but also an individual’s self-perception. Consequently, a person with dementia may avoid social situations, which can increase social isolation and contribute to social exclusion (Greenwood & Smith, 2016; Johannessen & Möller, 2011).

Researchers have used a range of approaches to study identity in the context of dementia (Caddell & Clare, 2010). In this article, we take a narrative approach, where narrative refers to the accounts of lived experience (Baldwin et al. 2008). Narrative construction can allow people with dementia to preserve, update and define their identity following the diagnosis (Hillman et al., 2018; Ryan et al., 2009). Consistent with this, people with dementia have narrated their lived experiences by writing books (e.g. Mitchell, 2019), speaking at conferences (Gilmour & Brannelly, 2010), educating students (Hope et al., 2007) and appearing in television programmes (e.g. Living with Dementia – Chris’ Story; David, 2016). By communicating their perspectives in this way, these individuals have been able to explore a positive identity with dementia and engage in a social dialogue that challenges stereotypical assumptions, thereby moving beyond traditional suffering narratives and affirming their identities as people who can live with the condition (Baldwin et al. 2008; Ryan et al., 2009).

The authors of these narratives tend to be younger people with atypical diagnoses, which have caused some individuals to question their legitimacy as people with dementia (e.g. Howard, 2017). In an analysis of autobiographies written by people with dementia, Page and Keady (2010) found that all the books they reviewed were written by people with young-onset dementia. They argued that the younger age of onset is particularly distressing and life-changing, and contributes towards a decision to publicly communicate one’s lived experiences. Page and Keady (2010) also found that authors felt they had a duty to communicate their lived experiences and ‘speak up’ on behalf of those who are not able to do so, with many recognising that they had a limited window of opportunity to share their perspectives.

One important vehicle through which narrative can be communicated is social media; yet, relatively little research has examined the use of social media by people with dementia. People with young-onset dementia may be more likely to use social media than those with late-onset dementia, given that in the general population younger people more frequently report using social media than older people (PEW Research Center, 2019). People with dementia have been found to use a range of social media, including forums, blogs and Facebook (Craig & Strivens, 2016; Kannaley et al., 2019; Rodriquez, 2013). Rodriquez (2013) found that people with early-onset Alzheimer’s disease used an online forum to narrate selfhood. By sharing stories, giving advice, offering encouragement and commiserating about their symptoms, forum members were able to construct a sense of community. In an analysis of blog narratives, Kannaley et al. (2019) found that people with dementia wrote about the effects of the disease, seeing the positives, feeling out of control, advocacy and empowerment, coping mechanisms and compensatory strategies, and candid descriptions of living with dementia. Similarly, Craig and Strivens (2016) analysed a Facebook group for people with young-onset dementia, finding that the group provided members with a unique opportunity for expression, support and awareness-raising. Importantly, these social media have allowed people with dementia to connect with others outside of their immediate networks and have provided an additional means of constructing narrative.

The microblogging site Twitter could be particularly valuable for people with dementia, as it has been shown to unite people who share a common goal and give voice to those who have been marginalised or ignored offline (Trevisan, 2017). Here, we view accounts that are communicated on Twitter as narratives. These narratives can be a powerful source of social change. The hashtag ‘#HelloMyNameIs’, for example, was a Twitter campaign that successfully raised awareness of the importance of communication in health care and influenced change in National Health Service (NHS) practices in the United Kingdom (Granger, 2013). Twitter could be equally valuable for people with dementia, providing a pathway to narrative and social inclusion.

The 280-character limit of tweets could also be beneficial for people with dementia when communicating lived experience. Other groups of people with disabilities have reported receiving additional benefits from Twitter compared with offline communication due to limitations in speech (Brunner et al. 2019; Hemsley et al., 2015). People with dementia may receive similar benefits from using Twitter due to difficulties with speech and concentration that can impede their communication in offline settings.

In a multipart study, we examined the use of Twitter by people with dementia over time (Talbot, 2020; Talbot et al., 2020a, 2020b). In the first phase of this study, we identified a population of people with dementia who use Twitter and, through a content analysis of their profiles, found that these users were relatively young and frequently identified themselves as advocates (Talbot et al., 2020a). In the second phase, we conducted a follow-up study with this population. We analysed tweets posted by a sample of these account holders and found that they used Twitter to engender a collective sense of identity through collective action, support and education, and communicated their individual identities by documenting lived experience (Talbot et al., 2020b). We also reported that the tweets of people with dementia were ‘short stories’ which formed an illness narrative over time. This is consistent with Thomas (2017) observations of two account holders with dementia, who used Twitter to develop and sustain social networks and provide vivid descriptions of their lived experiences. While this research has provided initial evidence on the value of Twitter for people with dementia, it has been predominantly informed by the tweets posted *by* people with dementia, and researchers have not yet directly engaged *with* people with dementia.

In this article, we address this gap in the literature and build on the earlier phases of our study by engaging with people with dementia. We aimed to amplify the voices of people with young-onset dementia and used a narrative model of identity to answer the following research questions:

(1) Why do people with dementia use Twitter?

(2) What challenges do people with dementia face when using Twitter?

## Method

### Design

This study was part of a larger multipart study that examined the use of Twitter by people with dementia over time (Talbot, 2020; Talbot et al., 2020a, 2020b). In this article, we present the findings of a thematic analysis of repeat interviews conducted with people with dementia about their use of Twitter.

### Participants

Participants were sampled from a population of Twitter users with dementia who we identified in the first phase of this study (Talbot et al., 2020a). All the account holders who identified themselves as living in the United Kingdom were tweeted by the first author, informing them of the research. The sample was limited to people living in the United Kingdom so that interviews could be conducted in person. The first author also searched Twitter to identify and contact additional account holders who may not have been identified in the first phase of this study. A snowballing approach was also used with existing participants and people with dementia who were advisors to the study. Inclusion criteria were as follows: self-identify as a person with dementia; live in the United Kingdom; be a user of Twitter; have capacity to give informed consent. Capacity was evaluated at the beginning of each meeting, using a support tool created by The Centre for Research in Ageing and Cognitive Health (REACH), University of Exeter. The first author assessed participants’ ability to understand and retain information about the research, to weigh up that information to reach a decision and to state a decision clearly.

The sample comprised 11 people with dementia, with eight identifying as men and three as women. This included one participant who dropped out of the study after the initial interview because his health deteriorated, but who consented to his data being used in this analysis. Participants were aged between 48 and 66 years (*M* = 59.73; *SD* = 6.05). All participants had been diagnosed before the age of 65 years, with age at diagnosis ranging from 45 to 63 years (*M* = 54.45; *SD* = 4.82). Participants had been living with a diagnosis of dementia for an average of 5.27 years (range:1–9 years; *SD* = 2.65). The most frequent diagnosis was mixed dementia (*n* = 4), followed by vascular dementia (*n* = 3), Alzheimer’s disease (*n* = 2) and posterior cortical atrophy (PCA; *n* = 1). One participant was unsure of her specific diagnosis. Table 1 describes the characteristics of the sample.

**Table 1.** Characteristics of the participants.

Pseudonym	Sex	Age	Age at diagnosis	Ethnicity	Previous occupation	Level of education	Type of dementia
Jack	M	66	63	White	School business manager	University degree	Mixed
Robert	M	65	57	White	Senior manager	NVQ (level 4)	Alzheimer’s
Susan	W	48	45	White	Nurse	University degree	Posterior cortical atrophy
David	M	65	56	White	Police officer	—	Vascular
Harry	M	57	51	White	Property manager	College education	Mixed
Paul	M	55	54	White	Clinical support worker	University degree	Mixed
Lewis	M	59	54	White	Local government officer	—	Vascular
Judith	W	59	53	White	Business support manager	University degree	Unknown
Richard	M	66	57	White	Lorry driver	—	Vascular
Heather	W	64	59	White	Horticulturist	—	Mixed
Fred	M	53	50	White	Business owner	—	Alzheimer’s

### Procedure

After ethical approval was obtained from the Human Research Ethics Committee of the University of Exeter Medical School (Reference: Oct17/B/126Δ1), participants took part in a series of interviews that explored their use of Twitter. After the initial interview, follow-up interviews took place six and 12 months later. All interviews were conducted in person by the first author at participants’ homes or a place of their choosing.

Each interview was split into two sections. In the first section, participants were asked about their experiences of using Twitter, with the discussions guided by a semi-structured interview guide. Interview guides were developed for each time point. Each interview guide was reviewed by two people with dementia to ensure relevant topics were being covered, questions were being asked in an appropriate and adaptable sequence and language was accessible. In the interviews, participants were encouraged to engage in reflective storytelling, in which they were asked to discuss their experiences of being diagnosed with dementia and the role of Twitter during this time. They were also asked about the benefits of Twitter, how they presented themselves on Twitter, interactions with other account holders and barriers to using Twitter.

The second section of each interview followed an adapted version of the scroll back method, which has previously been used with Facebook data (Robards & Lincoln, 2017). Participants were presented with a random sample of 10 tweets they had posted in the previous 6 months, including retweets. Random sampling facilitated discussion about a range of tweets that might not have otherwise been mentioned in the first part of the interview. The random selection process yielded tweets covering many interesting topics such as documenting experiences, interactions with other account holders, promoting campaigns and raising awareness.

Participants were asked to explain why they sent each tweet, what they hoped to achieve by posting the tweet, how they felt at the time they sent the tweet and what type of reaction the tweet provoked. Interviews were recorded using a digital device, transcribed verbatim and anonymised. Pseudonyms are used in this article to protect participants’ confidentiality. Interviews ranged in length from 35 to 80 min.

### Analysis

Interviews were analysed using Braun and Clarke’s (2006; 2013) approach to thematic analysis. A thematic analysis was conducted because we wanted to examine shared experiences across the sample. We used Braun and Clarke’s (2006) approach because it is suitable for research questions related to experiences, can be used to create detailed accounts of data and provides the theoretical flexibility to fully explore our interest in identity and answer our research questions.

Firstly, the authors immersed themselves in the data by reading anonymised interview transcripts or listening to audio recordings of the interviews. The research team then discussed initial coding ideas. The first author subsequently coded the entire dataset using QSR International’s NVivo 11 software. Following this, codes and relevant data extracts were collated and examined to identify initial themes across the data. These themes were then reviewed by the research team and revisions were made where appropriate. The next stage of the analysis was an iterative and reflective process, whereby the authors repeatedly returned to the raw data and coding to refine the themes until all authors agreed upon the finalised themes.

## Findings

We generated four themes from the data: (1) re-establishing identity; (2) communicating and preserving identity; (3) redefining dementia identities; (4) threats to identity. We outline these themes below and use anonymised quotes to illustrate the findings.

### Re-establishing identity

Participants reported losses and changes in their working, familial and social roles. In response, participants used Twitter to re-establish their sense of identity. We generated two related subthemes, showing how people with young-onset dementia re-established their identities through community membership and by regaining a sense of purpose.

#### Community membership

Participants reported using Twitter to combat the loss of identity that accompanied their diagnosis, by seeking out online communities of people with dementia. Power (2014) argued that there is a close relationship between identity and connectedness, and this was mirrored in participants’ statements. For example, Paul described his experience of turning to communities on Twitter after losing his working identity:

‘I went to work for the last time in September, then I was signed off work and I retired in February. But there was nothing there…you’re just left...so I thought I’d have a go on Twitter, and I found a lot of people in the same situation as me, and we started learning from each other…so it’s helped me to start finding my way again’ (Paul).

Community membership was important to participants, with three people referring to it as a ‘lifeline’. Narrating lived experience on Twitter enabled participants to identify with online communities of people with dementia. In turn, this created opportunities for social connection and peer support. Heather described her experience of receiving peer support after tweeting about her symptoms:

‘You can tweet this has happened and then you get all these things. Either ‘oh sorry to hear that. You have a better night tonight’ or somebody will tweet ‘yes this happens to me’. So, you know well that’s normal. It must be a regular occurrence...and so you say oh yes, it’s the dementia. I’m not going mad’ (Heather).

For Heather, community membership allowed her to see that others were facing similar challenges, which normalised her own experiences. This suggests that membership of communities on Twitter could help to counter some of the feelings of isolation and loss of identity that frequently accompany a diagnosis of dementia (Spreadbury & Kipps, 2019).

Membership of online communities also created opportunities for participants to increase their number of offline social connections. This included attending Dementia Engagement and Empowerment Project (DEEP) group meetings, attending and speaking at conferences, going to dementia cafés, getting involved in research and being on advisory boards. Participants explained that being active on Twitter opened ‘a new world’, and one participant said he would not have known about relevant information if he did not use Twitter:

‘If it wasn’t for Twitter nobody would have sent me a text message or knocked on my door or told me about anything. I wouldn’t have known about anything that I do now without Twitter’ (Lewis).

These findings suggest that Twitter acted as a ‘springboard’ for some people with dementia, providing opportunities for meaningful offline activities and interaction. Other researchers have written about the ‘shrinking worlds’ of people with dementia (Duggan et al., 2008), referring to a reduction in the number of places a person with dementia feels comfortable. In contrast, our findings suggest that Twitter might counteract the shrinking world effect, by providing people with opportunities to expand their social worlds.

#### Regaining purpose

Participants said they gained a sense of purpose from their use of Twitter, by supporting others, educating themselves and others about dementia and engaging in online advocacy. For example, one participant explained:

‘I’m not a do-it-yourself-er anymore, I’m far less a husband and father than I was, so this is concentrating on something I can do and that’s talking about dementia and knowing about dementia. It’s about giving yourself confidence, value, and a sense of purpose’ (Harry).

By tweeting about dementia, Harry was able to establish himself as an authority figure within the dementia community, thus developing a positive identity with dementia as an expert by experience. Participants likened these meaningful online activities to work; in fact, two participants referred to using Twitter as their ‘job’. Previous work has shown that meaningful activity can preserve dignity and a sense of identity in people with dementia (Roach & Drummond, 2014), and that increased self-worth is associated with ‘living well’ (Lamont et al., 2020). Our findings indicate that Twitter may provide opportunities for people with dementia to develop a positive sense of identity and engage in meaningful activities, thereby enhancing feelings of self-worth.

Some participants reported being introduced to dementia advocacy on Twitter, which lead to them engaging in advocacy work (both online and offline) and, in turn, fostered a sense of purpose. This reflects research showing that advocacy can provide people with dementia a way of regaining purpose and respect, and ‘fighting back’ against the effects of the illness (Weetch et al., 2020). Jack described his experience of becoming involved in advocacy work through Twitter:

‘Then having found some of those people and starting to receive links to documents and meetings and things. I then, that, that lead to my first real involvement with other activists’ (Jack).

Indeed, engaging with information on Twitter was an important first step for many participants in developing an identity as an advocate. Bartlett (2014) reported that advocacy provided people with dementia with a working identity – something people with young-onset dementia often lose post-diagnosis (Greenwood & Smith, 2016). Our findings indicate that Twitter may provide a meaningful pathway to advocacy for some people with young-onset dementia, which can provide a new working identity at a time of perceived loss.

### Communicating and preserving identity

Participants said they used Twitter to narrate lived experience, which was ‘empowering’ and ‘therapeutic’ for many reasons. Firstly, participants felt they could communicate better on Twitter than in offline settings because they could communicate at their own pace, check the wording of tweets and not be interrupted:

‘When you’re using Twitter, you don’t have to think as much as when you’re having a face-to-face conversation with someone, because when you’re having a face-to-face conversation with someone you’re frightened of making a mistake, whereas with Twitter you can think about what you want to do’ (Robert).

Other participants felt that Twitter provided a safe space for self-expression because their friends and family members did not use Twitter. This contrasted with participants’ use of Facebook, which constrained self-expression due to family members using this platform. For example, Susan explained that she could be her ‘authentic self’ on Twitter and document her experiences of dementia without worrying about how this would affect friends and family members:

‘With Twitter, it’s more open and I don’t really need to be so careful because I can say what I want. If I have a really crappy day I can be as open as I want to on Twitter, whereas on Facebook I have to be a bit cagey because my family don’t want to hear my bad days’ (Susan).

Participants also said the character limit of tweets facilitated self-expression. As Robert stated: ‘I can’t do Facebook because there’s too many. You put too many sentences together, whereas Twitter it’s to the point’. This participant reported problems with concentration and the character limit of tweets made it easier for him to communicate. These findings indicate that the short-text nature of tweets may provide an accessible means of constructing narrative for some people with dementia, compared with other social media platforms. This is important as the voices of these individuals might not otherwise be heard.

Participants said they narrated lived experiences on Twitter so they could look back at them in the future. Susan described her experience of using Twitter for reminiscence:

‘By the time next year comes I’ll have forgotten what I’ve done this year because that’s what’s happened, so it’s nice to read back’ (Susan).

Others likened tweets to a resource of information, which they hoped people would access after they were no longer able to use social media:

‘You’re creating a legacy for people to… I suppose a resource of information for people to access for long after I’m unable to carry on doing what I’m doing’ (Fred).

We interpret this as evidence of participants preserving aspects of themselves in their tweets, which may serve as a valuable source of information about lived experience and be a useful tool for prompting memories. This has important implications for reminiscence therapy, whereby social media posts could be integrated into therapy to evoke memories and stimulate mental activity.

Despite participants generally reporting that Twitter facilitated self-expression, they also described challenges related to their symptoms. For example, participants said they often made mistakes in their tweets, and would reread their tweets before posting them. One participant described her experience of making mistakes in tweets when she was struggling with her symptoms:

‘I sent a tweet this morning, the other day, and it was all gobbledegook because my brain wasn’t working very well, and I think ‘oh’ so I’ll have to delete the whole tweet’ (Susan).

Indeed, on days when participants faced increased difficulties with their dementia, using Twitter was an effortful process requiring considerable concentration. It is likely that if tweeting becomes too much effort for people with dementia, they will stop using Twitter, which some participants said would leave them feeling ‘lonely’ and ‘disconnected’. While it might be a relief for some people to stop using Twitter when it becomes too challenging, it could impact their social connectedness and sense of identity by removing one of their outlets for expression.

### Redefining dementia identities

Participants reported using Twitter to redefine what it means to be a person with dementia, by challenging stigma and misconceptions about the diagnosis. In fact, participants recognised that they were already challenging dementia stereotypes by simply being active on Twitter. For example, Robert explained: ‘People are surprised that you’re actually on social media’.

By showing that it is possible to live with dementia on Twitter and publicly identifying as a person with dementia in their profiles, participants sought to represent dementia in a more positive way:

‘People don’t realise the positive side, that you can still live, and you can live for quite a long time, depending on the dementia. So, I use it to educate and to change minds about things’ (Harry).

In turn, participants hoped this would change public perceptions, combat the shame that often accompanies dementia (Aldridge et al, 2019) and give hope to others with the diagnosis:

‘Having a Twitter account is one way of actually saying look, I’m not ashamed of having a diagnosis, and all the stigma around it is wrong’ (Judith).

‘The more people that come out, the more we’ll normalise it’ (Harry).

Hillman et al. (2018) theorised that dementia narratives constitute a strategy through which people with dementia shape wider discourses about the condition. This also appeared true for our participants, who used Twitter to redefine what it means to live with dementia – for both themselves and others with the diagnosis.

Another way in which participants sought to redefine dementia identities was through using Twitter to raise awareness and campaign for changes that would improve the lives of people with dementia. These individuals aimed to get others to view dementia and the needs of those living with the diagnosis differently, thereby changing perceptions. For example, participants often commented on a lack of support for people with young-onset dementia, so they used Twitter to challenge the idea that only those over the age of 65 years develop dementia and campaign for tailored support services:

‘Making people aware that there are young people with dementia that need help and support, and access to the right help and support, and not just when you reach 65. Life doesn’t just start at 65 for people with dementia’ (Robert).

Participants explained that Twitter allowed them to reach a wider audience and connect with politicians, clinicians, researchers, organisations, local NHS trusts and local police forces. Indeed, participants often referred to the potential of tweets to reach a vast audience, with one participant (Robert) stating: ‘if they’re telling one other person then that person tells someone, it’s just like a snowball effect you know, if you send out positive messages’. Thus, these participants not only used Twitter to alert others to their needs but also used the platform as a vehicle for social change through which they presented dementia in a more positive light.

### Threats to identity

Whilst participants’ experiences of using Twitter were mainly positive, they did describe some negative interactions that they had online. As a result of tweeting about their lived experiences, some participants attracted attention from trolls (i.e. people who post provocative and offensive messages designed to create conflict and distress; Buckels et al, 2014). One participant described his experience of being trolled:

‘He said you’re all mad…but it was terrible to call us mad. I just thought ah. You know, ‘can tell you’re all demented’. Because some people just they come on and say all people with dementia are lunatics’ (Robert).

Being trolled was upsetting for participants, and problematic given that research has shown that trolling can negatively impact mental health and well-being (O’Reilly et al. 2018). Moreover, researchers have found that stigma is prevalent in tweets containing the term ‘dementia’ (Oscar et al., 2017). Our findings suggest that people with dementia who use Twitter will likely be exposed to these stigmatising tweets, which could negatively affect their well-being and sense of identity.

Some participants said they received backlash from other account holders as a result of presenting themselves as people *living with* dementia rather than *sufferers* or *victims*. These account holders publicly challenged the diagnoses of participants, and the term ‘dementia doubters’ emerged from our interviews to refer to these individuals. One participant described her experience of responding to a dementia doubter:

‘I came back with something, and I said: ‘I think we’re all the true faces’. We are all true faces of dementia. Our stage in time, whether we’re at the beginning or the end. There’s no right or wrong, and that’s what I came back with. But when I see this it makes me so cross and quite angry’ (Susan).

These threats to identity caused participants to feel angry, frustrated and upset. As a result, some participants questioned the legitimacy of their diagnosis, with one participant stating that he felt like a ‘fraud’:

‘It has a negative effect on you because then you can start questioning. Have I got dementia? Am I being a fraud? It sends those questions through your mind’ (Paul).

Unfortunately, almost all participants reported being exposed to tweets that questioned their diagnosis. These findings indicate that while Twitter was generally a positive space for participants, it might sometimes be a hostile environment for people with young-onset dementia whose presentation is not consistent with more typical (late-onset) dementia.

While Twitter has potential to give voice to those who have been marginalised or ignored offline (Trevisan, 2017), this was not always true for participants, who often described instances when they felt ignored on Twitter and were therefore sceptical about the impact they were having. Paul, for example, reflected on a time when he tweeted about his experience of attending a course for people with dementia, hoping that his local council would provide something similar his area:

‘I started writing about that course to try and encourage [city] council and that to take it up, which has fallen on deaf ears’ (Paul).

Unfortunately, not feeling heard by other account holders was a common experience among participants, leaving them feeling discouraged, frustrated and ignored. Twitter might provide a platform for people with dementia to broadcast their perspectives; however, it does not necessarily mean that others are listening. Thus, online advocacy by itself might not be enough to change the lived experiences of people with dementia.

## Discussion

We used a narrative model of identity to examine why people with young-onset dementia use Twitter and what challenges they face when using the platform. Our findings suggest that Twitter provides opportunities for people with dementia to re-establish, redefine, communicate and preserve their identities. Despite these benefits, we also found that Twitter can be a hostile environment for people who live with young-onset dementia, who often experience threats to their identity from trolls and dementia doubters.

Importantly, our findings highlight the value of Twitter for promoting a sense of identity among people with dementia through community membership, instilling a sense of purpose and facilitating self-expression. Given a growing focus on non-medical interventions to help patients manage long-term conditions (e.g. social prescribing; Drinkwater et al., 2019) and recent policy initiatives that aim to improve the social connectedness of people who feel isolated (Department for Digital, Culture, Media, and Sport, 2018), people with dementia could be encouraged to engage with communities on Twitter. This could help them to adjust to the diagnosis, combat isolation and promote a sense of identity at a time of perceived loss.

We found that people with dementia experienced difficulties when using Twitter because of their symptoms, indicating that there is scope for it to be made more accessible for people with dementia. Everyday technologies, including Twitter, have been developed in a ‘hypercognitive society’ (Post, 2000) that emphasises a person’s rational thinking and memory, and therefore makes assumptions about cognitive ability. There is increasing pressure for offline spaces to be dementia-friendly (e.g. Alzheimer’s Society, 2013); however, online spaces should also be accessible for people with dementia. Designers and developers of Twitter could, therefore, consider working with people with dementia to modify the platform to make it more accessible for people with dementia. Researchers could also work with people with dementia to develop social media guidelines that provide instructions and advice on how to use Twitter.

Our findings raise questions about what constitutes narrative. Tweet narratives differ from other forms of narratives, such as books (e.g. Mitchell, 2019), as they are communicated in less than 280 characters. Page (2012) theorised that tweets are the equivalent of ‘short stories’ that people tell about themselves, and our findings support this assertion by showing that the short-text nature of tweets facilitated communication of lived experience. By facilitating narrative, Twitter could be a valuable tool to promote the social inclusion of people with dementia, allowing this group of people to exercise their right to a voice on issues that affect them and to facilitate social change. As people with dementia are using Twitter in this way, researchers may be able to use Twitter as an efficient source of patient and public involvement, which could be facilitated by hashtag chats such as #AlzChat or #DiverseAlz. This approach to patient and public involvement could be used to overcome challenges and costs associated with face-to-face engagement activities for people with dementia (Bethell et al. 2018; Iliffe, McGrath, & Mitchell, 2011).

## Limitations

Our research focused on people with young-onset dementia; therefore, these individuals are not representative of people diagnosed later in life, nor do they claim to be. The findings of the first phase of this study, however, suggest that the population of Twitter users with dementia is small and comparatively young (Talbot et al., 2020a), so the current sample does appear to be representative. It is also likely that the population of Twitter users with a diagnosis of dementia will increase in size as younger generations who are more engaged with social media age (Smith & Anderson, 2018). It is, therefore, valuable to understand how people living with dementia are using Twitter, the perceived benefits of using the platform and the challenges they face. This study provides an important foundation for future research on the social media needs and experiences of those diagnosed with dementia later in life.

The sample lacked diversity as participants were all White British and tended to have reasonably high socioeconomic status. It is likely that minority and vulnerable groups of people with dementia, who experience intersectional oppression and face additional stigma (Adelman, 2016; Berwald et al., 2016), will have different experiences of using Twitter. For example, these groups may face discrimination, targeted trolling and issues of inclusivity. In the future, researchers could adopt an intersectional approach and recruit more diverse samples to examine the experiences of different groups of people living with dementia who use Twitter.

An adapted version of the scroll back method (Robards & Lincoln, 2017) was used in this study because it was expected that tweets would act as stimuli for memories and stimulate further discussion. In practice, however, we found that participants sometimes did not remember what they were thinking or feeling at the time of posting the tweets. For other participants, discussing their tweets was quite repetitive or they felt tired when they got to this part of the study. Despite these challenges, there were cases where the scroll back method prompted interesting discussion and it was particularly useful for participants who found it difficult to stay on topic, providing something tangible to focus on. The use of the scroll back method in this research constitutes a novel contribution to the field by using this method with people with dementia. In future, we recommend researchers ask participants to scroll through their social media posts on a device or share social media posts that they feel are important with the researcher ahead of interviews, rather than researchers choosing the posts. In turn, this may promote agency among participants and stimulate insightful discussion.

The focus of this analysis was cross-sectional rather than longitudinal. Given the progressive nature of dementia, it is likely that the use of Twitter by people with dementia will change across the disease trajectory. For example, people with dementia might use Twitter more for advocacy in the early stages and more for peer support in the later stages. In the future, researchers could conduct longitudinal research with people living with dementia to elucidate this process.

## Conclusion

In conclusion, the findings of this research suggest that people with young-onset dementia used Twitter to re-establish, redefine, communicate and preserve their identities. These findings suggest that Twitter could be an important source of post-diagnostic support for people with dementia. Clinicians, non-profit organisations, carers, friends and family members could, therefore, consider introducing people with young-onset dementia to Twitter to help them cope with the diagnosis. However, there are some risks as Twitter was sometimes a hostile environment for people with young-onset dementia who did not present in a ‘typical’ manner, and their symptoms created technical difficulties when using the platform. In future, platform developers could work with people with dementia to make Twitter more accessible for this group.
